# Attitudes Toward Assisted Suicide and Life-Prolonging Measures in Swiss ALS Patients and Their Caregivers

**DOI:** 10.3389/fpsyg.2012.00443

**Published:** 2012-10-25

**Authors:** Ralf Stutzki, Ursula Schneider, Stella Reiter-Theil, Markus Weber

**Affiliations:** ^1^Clinical Ethics Support and Accompanying Research, University Hospital Basel/Psychiatric University Hospitals Basel, Institut für Bio- und Medizin Ethik, University of BaselBasel, Switzerland; ^2^Muskelzentrum/ALS Clinic, Kantonsspital St. GallenSt. Gallen, Switzerland

**Keywords:** ALS, motor neuron disease, quality of life, depression, end of life

## Abstract

**Objectives:** In Switzerland, assisted suicide (AS) is legal, provided that the person seeking assistance has decisional capacity and the person assisting is not motivated by reasons of self-interest. However, in this particular setting nothing is known about patients’ and their caregivers’ attitudes toward AS and life-prolonging measures. **Methods:** Data was retrieved through validated questionnaires and personal interviews in 33 patients and their caregivers covering the following domains: physical function according to the revised Amyotrophic Lateral Sclerosis Functional Rating Scale (ALSFRS-R), demographic data, quality of life, anxiety, depression, social situation, spirituality, burden of disease, life-prolonging, and life-shortening acts. **Results:** In patients the median time after diagnosis was 9 months (2–90) and the median Amyotrophic Lateral Sclerosis (ALS) FRS-R score was 37 (22–48). The majority of patients (94%; *n* = 31) had no desire to hasten death. Patients’ and caregivers’ attitudes toward Percutaneous Endoscopic Gastrostomy (PEG) and Non-Invasive Ventilation (NIV) differed. Significantly more patients than caregivers (21.2 versus 3.1%) stated that they were against NIV (*p* = 0.049) and against PEG (27.3 versus 3.1%; *p* = 0.031). Answers regarding tracheotomy were not significantly different (*p* = 0.139). Caregivers scored significantly higher levels of “suffering” (*p* = 0.007), “loneliness” (*p* = 0.006), and “emotional distress” answering the questionnaires (*p* < 0.001). Suffering (*p* < 0.026) and loneliness (*p* < 0.016) were related to the score of the Hospital Anxiety and Depression Scale (HADS) in patients. **Conclusion:** A liberal legal setting does not necessarily promote the wish for AS. However, the desire to discuss AS is prevalent in ALS patients. There is a higher level of suffering and loneliness on the caregivers’ side. A longitudinal study is warranted.

## Introduction

During the course of the disease, Amyotrophic Lateral Sclerosis (ALS) patients may suffer from depression, hopelessness, the feeling of loneliness, and loss of control (Rabkin et al., 2000, 2005; Albert et al., 2005; Olney and Lomen-Hoerth, 2005). In the terminal phase respiratory distress, anxiety, and other distressing symptoms may occur (Mandler et al., 2001). Given the suffering associated with the disease, some patients choose to decline life-prolonging measures such as Percutaneous Endoscopic Gastrostomy (PEG) and Non-Invasive Ventilation (NIV) and/or wish to hasten death (Ganzini et al., 1998; Veldink et al., 2002; Fang et al., 2008; Maessen et al., 2009). In an early study from Oregon, about 56% of all ALS patients considered physician-assisted suicide (PAS; legalized after 1997) during the terminal phase and 73% of caregivers and patients had similar attitudes toward PAS (Ganzini et al., 1998). In the Netherlands, during the 2000–2005 period 16.8% of ALS patients decided for euthanasia or PAS (Maessen et al., 2009), while in Sweden (where PAS is not legalized), ALS patients have a sixfold increased risk of committing suicide (Fang et al., 2008). Factors such as depression, hopelessness, loss of meaning, and purpose in life have been discussed to be associated with the wish to hasten death, whereas the contrary applies to religious faith and spiritual beliefs (Rabkin et al., 2000, 2005; Albert et al., 2005; Olney and Lomen-Hoerth, 2005). These findings are not consistent between different countries (Maessen et al., 2009). Moreover, there is a lack of longitudinal studies analyzing changes of these factors over time. It is also unclear whether the legal background in different countries influences patients’ attitudes toward assisted suicide (AS) as comparative studies are lacking.

In Switzerland, however, assistance in committing suicide by a physician or a lay person is not explicitly regulated by law, but article 115 of the Swiss Penal Code allows assistance in suicide provided that the person seeking assistance has decisional capacity and the person assisting – physician or lay person – is not motivated by reasons of self-interest. Based on this article, Swiss “right to die” organizations offer assistance to commit suicide (Fischer et al., 2008). A recent study from the City of Zurich revealed that between 2001 and 2004 “Dignitas or Exit Deutsche Schweiz” had facilitated a total of 421 cases of AS (Fischer et al., 2008). Amongst the patients, 60% had been non-residents in Switzerland emphasizing the problem of “suicide tourism.” Twenty-four percent of ASs were patients with “neurological disorders” including ALS. However, no information is available about the total number of ALS patients, their motivation and associated factors that had made them choose this exit strategy.

The objective of this study is to analyze patients’ and caregivers’ attitudes toward AS, life-prolonging measures as well as associated factors (e.g., depression, quality of life (QoL), loneliness, suffering, education, profession, family status, living situation, Amyotrophic Lateral Sclerosis Functional Rating Scale (ALSFRS, and time after diagnosis) in a country with a comparatively easy access to AS.

## Methods and Participants

### Participants

Swiss patients and their primary caregivers were recruited from a tertiary referral center (Muskelzentrum/ALS clinic) at the Kantonsspital St. Gallen. Patients and caregivers attended the ALS outpatient clinic on a regular basis (usually every 3 months). Patients and caregivers had to be at least 18 years old. Further inclusion criteria for patients were a diagnosis of definitive, probable, or probable laboratory supported ALS according to the revised El Escorial Criteria (Brooks et al., 2000). Patients were only eligible if they had been informed about their ALS diagnosis, disease progression, prognosis, and therapeutic options including PEG insertion and different forms of ventilation (e.g., NIV, tracheotomy). The discussion about PEG and ventilation was usually triggered by clinical decline that resulted in use of PEG or NIV. Both issues are part of ALS – specific advanced directives which are routinely applied during this discussion (Benditt et al., 2001). Patients who inquired about these interventions shortly after diagnosis were also eligible. The study was approved by the local ethics committee and all patients and caregivers gave written informed consent.

For this study inclusion criteria, exclusion criteria, variables, and statistical analysis were pre-specified. Data were retrieved through questionnaires and personal interviews during home visits. The data collection took place as soon as patients had been informed about life-prolonging measures. Interviews were carried out by a researcher trained in interview technique, experienced in pastoral care, and medical ethics, not involved in clinical care of the patients and their primary caregivers. Patients and caregivers filled in the questionnaires simultaneously. In case the patient was unable to complete the questionnaire by his/her own hand due to weakness, the interviewer completed the questionnaire according to the patient’s statements. The mean duration of the interview was approximately 60 min. After the interview, the primary caregiver/relative had the opportunity to clarify any issues that may have arisen during completion of the questionnaire. Specifically the following data and questionnaires were retrieved/applied:

#### Demographic data

The collection of data included age, sex, living situation, education, profession, and religious confession.

#### Numerical rating scale

(Eleven-point format; 0–10) asking the following questions: (1) What is your current QoL?; (2) How much are you suffering from your disease/from the disease of your partner?; (3) How lonely do you feel?; (4) How strong is your current desire to ask others for help to end your life prematurely?; (5) How distressing or how helpful was it for you to speak about such issues?

#### Hospital anxiety and depression scale (HADS)

A self-assessment scale to quantify patients’ anxiety and depression by choosing one response from four given. The range is from 0 to 42 with the maximum score indicating a high level of depression and anxiety (Bjelland et al., 2002).

#### Questions regarding life-prolonging measures and hastening death

Patients’ and caregivers’ opinions were assessed with regards to tracheotomy, NIV, and PEG within a four-point response format. The following questions were asked: What is your attitude toward the following life-prolonging measures: (a) Tracheotomy; (b) NIV; (c) PEG? Possible answers to each item were: (a) I am not sure; (b) I am absolutely in favor of it; (c) I am in favor of it under certain circumstances; (d) I am against it.

Questions in yes/no format included: (1) Have you ever thought about committing suicide after receiving your diagnosis?; (2) Can you imagine a future scenario in which a physician prescribes a fatal drug which you administer yourself?; (3) Can you imagine a future scenario in which a physician prescribes and administers to you a fatal drug?; (4) Have you ever discussed suicide with others?; (5) Would you like to discuss suicide with a physician?

#### Idler index of religiosity (IRR)

The IRR assesses both public and private religiosity: (1) summing up attendance at religious meetings and services and the number of church members known to the patient; (2) self-assessment of personal religiosity as well as the amount of strength and comfort provided by personal faith (Robbins et al., 2001).

### Statistical analysis

For continuous variables (e.g., age) the mean of the differences between “patient” and “caregiver” was calculated by the *t*-test. Variables applying scores were compared by the Wilcoxon signed rank tests providing the median difference and its 95% confidence limits. Differences of ordered categorical variables (four-point response format) were tested by the Fishers exact test and the McNemar’s Chi-squared test. For analyzing possible associations between the paired samples (“patient,” “caregiver”) the Spearman’s rho correlation coefficient was applied. The level of significance was *p* < 0.05.

In order to predict score ratios between patients and caregiver, generalized linear mixed-effects models with group (“patient” and “caregiver”) and given variables as fixed factors (sex, age, education, profession, family status, living situation, children, ALSFRS, time after diagnosis, and QoL) and subject (“patient”) as random factor were performed either as multivariate or univariate model (for each parameter as a separate model). In order to predict dichotomous variables (yes versus no) concerning suicidal ideation for patients, logistic regression models were performed providing odds ratios (OR) and 95% confidence intervals (CI) with corresponding *p*-values.

All analyses were performed using R version 2.12.2 (R Development Core Team, 2011).

## Results

### Demographic data

During the recruitment period from 2008 to 2010 a total of 59 patients and caregivers were asked whether they would participate in this study. Twenty-six patients declined, 33 patients and their caregivers agreed to participate. The most frequent reason for declining participation was “no interest” and reluctance toward the themes of religiosity and spirituality. Table 1 summarizes the epidemiological data, social status, and religious denominations of patients and their caregivers. Mean age of patients was 59.6 and mean age of caregivers was 56.9 (paired *t*-test; *p* = 0,065). The median time after diagnosis at which the first interview took place was 9 months (2–90) and the median ALSFRS-R was 37 (22–48).

**Table 1:** **Demographic data**.

	Patients % (*n*)	Caregivers % (*n*)
Age (mean, range)	59.6 (38–79)	56.9 (31–79)
Sex		
Female	36.4 (12)	62.5 (20)
Male	63.6 (21)	37.5 (12)
Living situation		
Alone	9.1 (3)	3.1 (1)
With spouse	57.6 (19)	59.4 (19)
With spouse and child(ren)	33.3 (11)	37.5 (12)
Religious confession		
Roman-catholic	51.5 (17)	46.9 (15)
Protestant	33.3 (11)	25.0 (8)
No confession	15.2 (5)	28.1 (9)

### Assisted suicide/hastening death

Thirteen patients (39%) answered that during the course of the disease they had thought about the possibility of committing suicide (Table 2). However, at the time of the interview, 31 of the patients (94%) expressed no wish to hasten death by AS. Thirty-three percent of the patients would like to discuss the issue “suicide” with a physician. Fifty-four percent of the patients could imagine asking a physician in the future to prescribe a fatal drug that they could take themselves; 57% could imagine a physician administer such a drug to them in the future. Logistic regression revealed that for patients QoL was the major predictor toward suicidal ideation (OR: 0.58, CI: 0.35–0.99) and the wish to discuss suicide with a physician (OR: 0.32, CI: 0.13–0.81, Table 2). The number of children (OR: 0.54, CI: 0.28–1.04) and HADS score (OR: 1.2, CI: 0.99–1.45) was also predictive of the wish to discuss suicide with a physician. Other analyzed factors (education, profession, family status, living situation, ALSFRS, and time after diagnosis) were not associated.

**Table 2:** **Patient suicidality; n.s., no significant effect**.

	Yes % (*n*)	No % (*n*)	No answer % (*n*)	Associated factors (adjusted for gender and age)
Thought about suicide after receiving diagnosis	39.4 (13)	60.6 (20)	0	n.s.
Can imagine future scenario: committing suicide by means of a prescribed drug	54.5 (18)	45.5 (15)	0	Quality of life (*p* = 0.026)
Can imagine future scenario: suicide with the help of physician administering fatal drug	57.6 (19)	42.4 (14)	0	n.s.
Have already discussed suicide with others	33.3 (11)	66.7 (22)	0	n.s.
Would like to discuss suicide with a physician	33.3 (11)	60.6 (20)	6.1 (2)	Number of children (*p* = 0.048), quality of life (*p* < 0.001), HADS (*p* = 0.037)

### Life-prolonging measures

The majority of patients (57.6%) and caregivers (50.0%) were against tracheotomy (Table 3). As verified by the McNemar test no significant difference was detected between the coincident answers of the two study groups (*p* = 0.37). No patient and no caregiver were generally in favor of its application, only “under certain circumstances” (27.3 patients versus 25.0% caregivers). The remaining interviewees were “unsure” about tracheotomy. Within the four-point response format attitudes between patients and caregivers regarding NIV and PEG differed (Fishers exact test). Significantly more patients than caregivers (21.2 versus 3.1%) stated that they were against NIV (*p* = 0.049) and against PEG (27.3 versus 3.1%; *p* = 0.031).

**Table 3:** **Live-prolonging measures: “what is your attitude toward the following life-prolonging measures?” *p*-values derived from McNemar test**.

	Not sure% (*n*)	Absolutely yes % (*n*)	Yes under certain circumstances % (*n*)	Against % (*n*)	*p*
Tracheotomy					0.37
Patients	15.2 (5)	0	27.3 (9)	57.6 (19)	
Caregivers	25.0 (8)	0	25 (8)	50.0 (16)	
NIV					0.17
Patients	3.0 (1)	42.4 (14)	33.3 (11)	21.2 (7)	
Caregivers	18.8 (6)	43.8 (14)	34.4 (11)	3.1 (1)	
PEG					0.75
Patients	12.1 (4)	24.2 (8)	36.4 (12)	27.3 (9)	
Caregivers	25.0 (8)	18.8 (6)	53.1 (17)	3.1 (1)	

### Quality of life and burden of disease

The median of QoL rated on a 11-point scale was six for patients and caregivers (*p* = 0.68). “Suffering,” “loneliness,” and “emotional distress answering the questionnaire” were significantly higher on the caregivers’ than on the patients’ side (Table 4). The mean HADS score of patients was 10.6 ± 5.1. Univariate analysis by a general linear mixed-effects model revealed a significant influence of the HADS on “suffering” (OR: 1.04 (95% CI: 1.01–1.07, *p* = 0.027) and “loneliness” (OR: 1.17 (95% CI: 1.04–1.33, *p* = 0.017) for coincident answers of patients and caregivers. Other analyzed factors (sex, age, education, profession, family status, living situation, children, ALSFRS-R, time after diagnosis, and QoL) did not show a significant effect, both in univariate and multivariate analysis (data not shown).

**Table 4:** **Quality of life and burden of disease variables rated on self-rating scales (0–10)**.

Parameters	Patients (median, IQR)	Caregivers (median, IQR)	Difference of medians	Lower 95% CI	Upper % 95 CI	*p*-value
Quality of life	6 (5–8)	6 (5–7)	0.25	−1.0	1.5	0.68
Loneliness	0 (0–1)	2 (0–6)	−3.25	−5.5	−1.5	0.003
Emotional distress	0 (0–1)	3 (0.8–5)	−4.25	−5.5	−3.0	<0.001
Suffering	5 (3–6)	6.5 (5–8)	−2.25	−3.5	−0.5	0.006

*Differences determined by Wilcoxon signed rank test. IQR, inter quartile range*.

### Religiosity

With regard to publicly practiced or private religiosity patients considered themselves to be more religious than their caregivers (*p* < 0.001) and derived more strength and comfort from their faith (*p* < 0.01).

## Discussion

The most important finding of the study is that at the time of the interview 94% of the patients had no intention to hasten death. This seems notable as the Swiss legal situation is liberal regarding assistance to commit suicide and the society is tolerating the practice of lay organizations offering the assistance through the collaboration of physicians and lay persons (van der Heide et al., 2003; Reiter-Theil, 2006). In contrast, a comparable study from Germany on ALS patients revealed that 37% of patients wished to hasten death (Jox et al., 2007). Germany is characterized by a liberal regulation of AS in penal law, but at the same time by a restrictive regulation and prohibition of PAS in the medical law (Reiter-Theil, 2006). The only difference between the Swiss and the German study methodology was that our patients were interviewed at home, whereas in the German study the interviews took place in the outpatient clinical. This difference, however, is unlikely to account for the imbalance and suggests that not the legal background as such (e.g., a more liberal legal situation promotes AS), but other factors must be responsible for the wish to hasten death. Previous studies from Oregon and the Netherlands where PAS is also legal, revealed a high prevalence of AS and euthanasia among ALS patients (Ganzini et al., 1998, 2002; Veldink et al., 2002; Albert et al., 2005; Maessen et al., 2009). This contrasts with our findings where only a minority of patients expressed a wish to hasten death. However, a comparison of our results with the Oregonian and Dutch studies is difficult as they were either retrospective (Veldink et al., 2002; Maessen et al., 2009) or interviews took place at a late to terminal stage (Ganzini et al., 2002; Albert et al., 2005). Longitudinal studies analyzing attitudes toward AS have not been published yet, but are important to understand whether these attitudes depend on the degree of disability.

With respect to preferences for life-prolonging and ameliorative technologies it seems that ALS patients make choices consistent with preferences expressed shortly after diagnosis (Albert et al., 1999). It is unclear whether this also applies to attitudes toward AS. Nevertheless, more than one-third (39%) of our patients had thought about the possibility of committing suicide after being diagnosed with ALS and 33% of the patients expressed the wish to discuss suicide with their physician. Even 58% said they could imagine a future scenario in which a physician would not only prescribe, but also administer a fatal drug (i.e., euthanasia which is prohibited in Switzerland) to them. This mirrors other studies which have shown that the themes of assisted dying and suicide are prevalent in ALS patients (Ganzini et al., 1998, 2002; Albert et al., 2005; Palmieri et al., 2010). The wish of patients to discuss suicide with a physician was associated with poorer QoL and a higher HADS score. It can be concluded that medical caregivers need to develop an openness and willingness to discuss with and inform patients about suicide and to deal with the fact that a considerable number of patients facing end of life may ask for their physician’s active involvement in hastening death (Bascom and Tolle, 2002; Jox et al., 2007; Moore et al., 2007; Oliver et al., 2007). The prevalence for a physician’s active involvement in hastening death in the future corresponds with a retrospective study (Lofmark et al., 2008) which reports that 37% of the Swiss physicians (*n* = 1397) had received a patient request to hasten death.

Patients’ and caregivers’ attitudes regarding life-prolonging measures were largely concordant. Fifty-eight percent of patients and 50% of caregivers were against tracheotomy. However, caregivers (mean: 22.2%) in general were considerably more unsure about NIV, PEG, and tracheotomy than patients (mean: 9.8%). Furthermore significantly more patients than caregivers were strictly against PEG and NIV. This confirms a fundamental need for more information and discussion about life-prolonging measures as has also been shown by other studies (Albert et al., 1999; Trail et al., 2003).

Another important finding of this study is that caregivers scored significantly higher in the domains of suffering, loneliness, and distress filling out the questionnaires compared to patients, despite overall QoL being not different between patients and their caregivers. The only associated factor was patients’ depression and anxiety as measured by HADS. Sex, age, education, profession, family status, living situation, children, ALSFRS, time after diagnosis, and QoL were not related. Previous studies also revealed that depression in patients is associated with caregiver burden (Chio et al., 2005) and that patients’ and caregivers’ well-being are interrelated (Pagnini et al., 2011). Other examined factors such as time since diagnosis and degree of disability were not related in our as in previous studies (Rabkin et al., 2000; Chio et al., 2005). This is in contrast with studies that have shown that caregiver burden and distress are associated with the degree of disability (Hecht et al., 2003; Chio et al., 2005; Lo Coco et al., 2005). However, it needs to be taken into account that the patients in our study had only mild to moderate handicap as compared to other studies. A prospective longitudinal study may help to resolve this discrepancy and would also reveal dynamic changes as disease and disability progress.

This study has several limitations. Patients were recruited from a single center and may therefore not be representative for all Swiss ALS patients. At our ALS Clinic patient care is strictly adhering to international guidelines (Miller et al., 2009; Andersen et al., 2012) which may not be the case in all Swiss centers. Each follow-up consultation lasts 2 h allowing ample time to discuss end of life decisions and related issues that may have influenced the results. Second, since spirituality and religiosity were part of the study, this may have introduced a self-selection bias toward couples that tend to be more religious which is known to be inversely related to the wish to hasten death (Albert et al., 2005). The themes of religiosity/spirituality may also contribute for the relatively high non-participate rate of 44%. However, these problems are inherent in almost all of the published interview studies.

## Conclusion

In summary the “liberal” Swiss legal setting does not promote the wish for AS, but the wish to discuss AS is prevalent amongst ALS patients even in moderately advanced stages of the disease. This wish is associated with poorer QoL and degree of depression.

## Conflict of Interest Statement

The authors declare that the research was conducted in the absence of any commercial or financial relationships that could be construed as a potential conflict of interest.

## Author Note

This study was approved by the ethics committee of St. Gallen.
